# *MoleCoolQt* – a molecule viewer for charge-density research

**DOI:** 10.1107/S0021889810042482

**Published:** 2010-11-27

**Authors:** Christian B. Hübschle, Birger Dittrich

**Affiliations:** a Institut für Anorganische Chemie, Georg-August-Universität Göttingen, Germany

**Keywords:** *MoleCoolQt*, charge density, visualization software

## Abstract

*MoleCoolQt* is a molecule viewer for charge-density research. Features include the visualization of local atomic coordinate systems in multipole refinements based on the Hansen and Coppens formalism as implemented, for example, in the *XD* suite. Residual peaks and holes from *XDfft* are translated so that they appear close to the nearest atom of the asymmetric unit. Critical points from a topological analysis of the charge density can also be visualized. As in the program *MolIso*, color-mapped isosurfaces can be generated with a simple interface. Apart from its visualization features the program interactively helps in assigning local atomic coordinate systems and local symmetry, which can be automatically detected and altered. Dummy atoms – as sometimes required for local atomic coordinate systems – are calculated on demand; *XD* system files are updated after changes. When using the invariom database, potential scattering factor assignment problems can be resolved by the use of an interactive dialog. The following file formats are supported: *XD*, *MoPro*, *SHELX*, *GAUSSIAN* (com, FChk, cube), CIF and PDB. *MoleCoolQt* is written in C++ using the Qt4 library, has a user-friendly graphical user interface, and is available for several flavors of Linux, Windows and MacOS.

## Abstract 2

*MoleCoolQt* is a molecule viewer designed for experimental charge density studies with a user-friendly graphical user interface.

## 1. Introduction

Visualization is an important aid to the scientific understanding of nature. Today’s users often expect a user-friendly and intuitive graphical user interface (GUI). Achieving user friendliness is a difficult task, because user preferences might differ considerably. In the author’s opinion, a good tool for a wider scientific audience should help in ‘fixing screws’ but should not lead to a black box where the screws are hidden away.

There are many molecule viewers available, for example *Coot* (Emsley *et al.*, 2010 ▶), *Mercury* (Macrae *et al.*, 2006 ▶, 2008 ▶), *DRAWxtl* (Finger *et al.*, 2007 ▶) and *OLEX2* (Dolomanov *et al.*, 2009 ▶), each of them focusing on a different research area, such as macromolecular modeling or small-molecule refinement. *MoleCoolQt* is a molecule viewer that helps researchers in the charge-density field in preparing input files and in producing eye-catching visualizations of results.

*MoleCoolQt* provides three-dimensional graphical visualization of molecular structures from single-crystal X-ray diffraction together with a visualization of the local coordinate system, which is needed for multipole refinement. A wrong assignment of local atomic coordinate systems is a common source of error in refinement. Likewise, over-parameterization is another potential source of ill-refined multipole parameters, and it can be avoided by following the program’s suggestions of local atomic site symmetry.

## 2. Technical description and functionality

**Figure 1:**
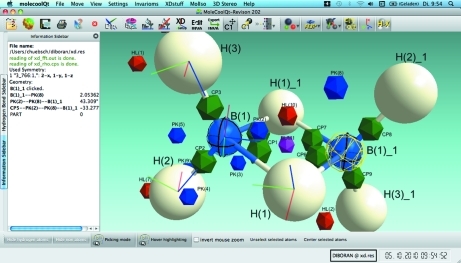
The main window with the structure of β-diboran (Hübschle *et al.*, 2004 ▶) loaded. Symmetry-generated atom B(1)_1 is selected. Bond critical points are shown as green icosahedra. Residual peaks and holes are visualized as blue and red icosahedra. Dummy atom ‘DUM1’ (pink icosahedron) is used for the definition of the local atomic coordinate systems of B(1) and H(1). Short blue, red and green lines are local atomic coordinate systems (*x*, *y* and *z*).

The program GUI (Fig. 1 ▶) is based on the widely used Qt4 library, while the graphical functionality is based on OpenGL. *MoleCoolQt* is written in C++ and is fully portable. It runs under Linux, Windows and MacOS Snow Leopard platforms. The GUI is menu-oriented and can be controlled by mouse and keyboard short cuts. Unlike other programs – *e.g. OLEX2* (Dolomanov *et al.*, 2009 ▶) – *MoleCoolQt* does not have a command line.

### 2.1. *XD* setup dialog

**Figure 2:**
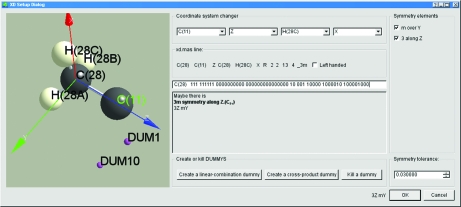
The *XD* setup dialog window.

Calculating dummy atoms in the *XD* suite (Volkov *et al.*, 2006 ▶) is usually a laborious and time-consuming task. *MoleCoolQt* provides an interactive *XD* setup dialog (Fig. 2 ▶), which can automatically find local symmetry and set up on the fly the corresponding lines in the *XD* master file. The extent of local atomic symmetry can be controlled by check boxes. Dummy atoms can be generated by a few clicks in the same dialog.

### 2.2. *XD* master file editor

The *XD* setup dialog described in the previous section is a tool to help edit the *XD* master file. Experience teaches that even the most intelligent tool cannot replace user choice and input. Therefore, the text editor function in *MoleCoolQt* provides syntax highlighting. The user can manipulate the master file directly without getting lost in command syntax. Syntax highlighting aids in finding wrong input.

### 2.3. Disordered structures

When working with *SHELX* (Sheldrick, 2008 ▶) files *MoleCoolQt* considers ‘PART’ instructions for drawing bonds for disordered groups. Unfortunately, in *XD* there is no expression for ‘PART’. If *SHELX* input files are present in the working directory of an *XD* refinement, then ‘PART’ information is extracted and a human-readable auxiliary file (xd_part.aux) is written. The xd_part.aux file is then used for bond generation and for invariom assignment (see §[Sec sec2.4]2.4).

### 2.4. *InvariomTool* name-assignment helper

For the application of nonspherical scattering factors (invarioms) from the invariom database (Dittrich *et al.*, 2006 ▶) the functionality of the preprocessor program *InvariomTool* (Hübschle, Luger & Dittrich, 2007 ▶) is used. Automatic scattering factor assignment can fail in cases where bond distances differ strongly from expected values. In such cases *MoleCoolQt* provides interactive dialogs to check if the scattering factors found are present in the database and to help reassign these by changing the bond order.

Input files for invariom refinement including database multipole parameters and modified local atomic coordinate systems can be generated for both the *XD* (Volkov *et al.*, 2006 ▶) and the *MoPro* (Jelsch *et al.*, 2005 ▶) packages.

### 2.5. Molecular isosurfaces

The program *MolIso* (Hübschle & Luger, 2006 ▶) can visualize color-mapped isosurfaces from *XD* grid files and *GAUSSIAN* (Gaussian Inc., Pittsburgh, PA, USA) cube files. It is controlled *via* an initialization file and a context menu. Because it might be difficult for an unexperienced user to work with an ‘ini’ file and because of other shortcomings of the GLUT-based GUI (*GLUT – The OpenGL Utility Toolkit*; http://www.opengl.org/resources/libraries/glut/) in *MolIso*, the functionality of *MolIso* was built into the GUI of *MoleCoolQt*. New features and improvements over *MolIso* include the following:

(1) Freely scalable labels with all system fonts are available.

(2) Screenshots can be produced with up to fourfold screen resolution for the generation of high-quality illustrations.

(3) The number of different isovalues and hence the number of isosurfaces that can be displayed simultaneously is now essentially unlimited.

(4) The legend is scalable, movable and can be oriented vertically or horizontally.

(5) The color gradient used for the visualization is configured *via* a dialog at startup.

(6) The orientation of the molecule, the legend size and position, and the contour line width and density can be saved in a file to produce a series of pictures.

### 2.6. Displacement ellipsoids

**Figure 3:**
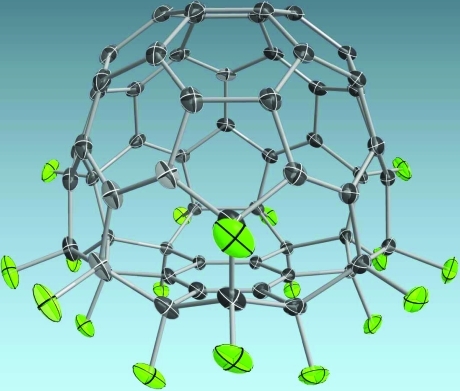
The crystal structure of C
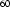
F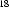 (Hübschle, Scheins *et al.*, 2007 ▶) with displacement ellipsoids at the 70% probability level. The visualization of semi-transparent displacement ellipsoids with intersecting planes and principal ellipses is illustrated.

Atomic displacement parameters can be visualized as displacement ellipsoids (Fig. 3 ▶). Intersecting planes, principal ellipses and transparent ellipsoids can be customized for each element in the atom parameters dialog. In this dialog, covalent radii, ball size in ball-and-stick mode and element color can also be set.

### 2.7. Other visualization features

When loading a structure from an *XD* multipole refinement, *MoleCoolQt* searches for auxiliary *XD* files in the same directory. One of the files is xd_fft.out, written by the program *XDfft*. The Fourier peaks and holes are extracted from that file and are visualized as blue and red icosahedra translated *via* symmetry operations so that they appear close to the atoms of the asymmetric unit.

When a critical-point search is carried out by the program *XDprop*, a file xd_rho.cps is written. If this file is present, *MoleCoolQt* represents critical points as green icosahedra. The user can get information about the electron density or peak height by clicking on these objects when the program is in ‘picking mode’.

Dipole moments given in a user-generated text file can be loaded into the molecular representation. They are visualized as thin arrows originating at the molecular center of mass.

The program can also provide stereographic projection modes, including one for Zalman monitors. For these monitors, glasses for current (RealD) three-dimensional cinema movies can be used and there is no need for a special hardware driver. The source code for this feature was taken from the program *Coot* (Emsley *et al.*, 2010 ▶).

### 2.8. Packing capabilities

When crystallographic structure files are loaded, molecules can be packed to fill a unit-cell box, or within a freely selectable distance from the asymmetric unit. By clicking (right mouse button) on an atom, the structure representation can be expanded around this atom. By default a molecule is completed if only a part of it is within the asymmetric unit. In the case of *XD* files the local atomic coordinate systems are shown for the asymmetric unit only.

### 2.9. Geometry calculations

Clicking a series of atoms or other objects leads to feedback on distances, angles and torsion angles of the selected objects in a dockable information window. Dockable means that the window is normally part of the main window, but can be detached by the user to move it somewhere else on the screen. There is another dockable side window like this where hydrogen bonds are reported in a table.

## 3. Example

**Figure 4:**
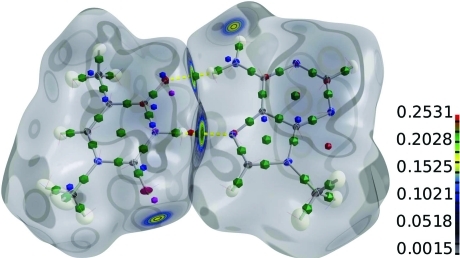
Representation of the Hirshfeld surfaces of the Hoogsteen base pair 1-meth­yl­thymine and 9-methyladenine, color mapped by the electron density. Green icosahedra are bond and ring critical points; blue and red icosahedra are residual density peaks and holes; purple icosahedra are dummy atoms for local atomic coordinate systems.

Fig. 4 ▶ illustrates some of the capabilities of *MoleCoolQt*. The electron density from an experimental multipole refinement is color mapped onto the Hirshfeld surfaces of the Hoogsteen (1963 ▶) base pair 1-meth­ylthymine and 9-methyladenine.

Hirshfeld surfaces were introduced by Spackman & Byrom (1997 ▶) (see also McKinnon *et al.*, 1998 ▶), and their representation was originally implemented in *CrystalExplorer* (Wolff *et al.*, 2010 ▶). For the picture shown here, two grid files were generated by *XDprop*. The first file contained the independent atom model electron density of the molecule and the second that of the molecule surrounded by its neighbors. To obtain the Hirshfeld surface, both were divided using an external Perl script.

A color gradient is combined with different degrees of transparency depending on the magnitude of electron density in order to focus attention onto regions of hydrogen bonding.

## 4. System requirements

*MoleCoolQt* is developed under SuSE Linux 11.2. Executables are available for SuSE 11.1, 11.2 and 11.3, each for 32 and 64 bit, and are generated automatically every day *via* a script using the Subversion version-control system. An Ubuntu version can also be provided. We intend to maintain the code for future Linux versions and other distributions. The Windows version has been tested under Windows XP, Vista and 7. The version for MacOS X is available as a compressed disk image file (.dmg) and has been tested to run under Leopard (10.5) and Snow Leopard (10.6).

## 5. Online help and documentation

At present the detailed documentation is still work in progress. However, for many features in *MoleCoolQt* a ‘What is This’ help is implemented. Clicking the ‘What is This’ is question mark button starts the help mode. After clicking on a specific item on the GUI in this mode, a short help window is displayed. In addition, help on a few topics can be found in the online forum at http://www.molecoolqt.de. At this site one can also find some video tutorials.

## 6. Licensing, availability and future development

*MoleCoolQt* is licensed under the Lesser General Public License. It can be downloaded free of charge at http://www.molecoolqt.de. Registration to the forum at this site is not required but is encouraged to keep users informed about new versions and new features. The program is still under development so that new features might be added or other features optimized further. For the near future it is planed to add more features to the *MolIso* part of *MoleCoolQt*.
